# Inhibition of Intestinal Cellular Glucose Uptake by Phenolics Extracted from Whole Wheat Grown at Different Locations

**DOI:** 10.1155/2018/5421714

**Published:** 2018-03-18

**Authors:** Maryam Shamloo, Peter J. H. Jones, Peter K. Eck

**Affiliations:** ^1^Richardson Centre for Functional Foods and Nutraceuticals, University of Manitoba, Winnipeg, MB, Canada R3T 2N2; ^2^Department of Food Science, University of Manitoba, Winnipeg, MB, Canada R3T 2N2; ^3^Department of Human Nutritional Sciences, University of Manitoba, W569 Duff Roblin Building, 190 Dysart Road, Winnipeg, MB, Canada R3T 2N2

## Abstract

Whole grain consumption is associated with reduced risk of type 2 diabetes, and the underlying mechanism might be related to the actions of polyphenols. Dietary polyphenols contribute to low glycemic indices through inhibition of intestinal glucose transport proteins. This study has two objectives: (1) to evaluate how the contents of phenolic acids in wheat vary by genetic background and growth condition and (2) to evaluate how these changes translate into physiologic relevance by investigating cellular glucose transporter inhibitions. Phenolic acids were extracted from wheat varieties grown at different locations over two crop years. The degree of inhibition of glucose uptake into human Caco-2E cells was determined. Free and bound phenolic acid extracts of all wheat genotypes inhibited glucose uptake. Degree of glucose uptake inhibitions positively correlated with the contents of free and bound phenolic acids, and the correlation coefficients were *R*^2^=0.91 and *R*^2^=0.89, respectively. Genotype and environment influenced the content of free and bound phenolic acids which linearly translated to the degree of glucose uptake inhibition in a model of intestinal absorption (*P* < 0.05). Results of this work mechanistically support the hypothesis that dietary phenols positively influence the glycemic index and therefore the health properties of whole grain consumption.

## 1. Introduction

Diabetes mellitus has become an emerging global health problem. Currently, estimated 422 million adults are suffering from diabetes and that number will increase to 642 million by 2040 [1]. Type 2 diabetes is characterized by the body's insensitivity to insulin and is characterized by increased blood sugar levels [2]. In diabetic patients, the expression of intestinal glucose transporters has been reported to be 3- to 4-fold higher than healthy controls [3], and therefore, higher amount of glucose will be absorbed by these patients in a shorter period of time, leading to increased postprandial glycemia. Thus, an effective treatment option for diabetes and diabetes-related complications is to dampen or inhibit intestinal glucose transporters and/or glucose absorption.

Whole wheat is the main ingredient in a range of staple foods that form a part of a healthy diet [4]. Regular intake of whole grain products is associated with a 20–30% reduction in the risk of type 2 diabetes [5, 6]. For example, whole wheat breads generate lower glycemic indices than white bread [7], which is in part attributed to polyphenols found in the bran fraction of whole wheat [8]. These polyphenols are known to be efficient in lowering blood glucose levels [9]; however, the mechanisms remain largely undetermined [6]. Some plant bioactives inhibit intestinal glucose transporters [10, 11] causing a blunted glucose absorption [6, 7, 12].

Wheat genetics and growing environments play a critical role in influencing the accumulation of secondary plant metabolites [13], including polyphenols. The importance of the genotype is demonstrated by the fact that winter wheat varieties contain twice the levels of total phenolic acids than other genotypes [14]. The impact of the environment is very distinct on levels of free phenolic acids; for example, the levels of free phenolic acids in 26 wheat genotypes grown in Hungary in three consecutive crop years were largely influenced by the effect of environment. On the other hand, levels of bound phenolic acids were stable throughout the growing seasons and were mostly determined by genetics [15].

In earlier studies under strictly controlled growth conditions, we had observed strong positive correlations between growing temperatures, levels of phenolic acids, and the inhibition of glucose uptake into Caco-2 cell monolayers exhibited by extracts from whole wheat (unpublished data). Here, we investigated if such correlations can be observed for wheat grown under field conditions.

## 2. Materials and Methods

### 2.1. Wheat Genotypes

Eight western Canadian wheat genotypes (*Triticum* spp.), representing different commercial classes with different qualities, including AC Corrine, AC Barrie, AC Crystal and Carberry, Snowbird, AC Andrew (*Triticum aestivum* L.), AC Navigator and Strongfield durum wheat grains (*Triticum turgidum* L. var. durum). These eight genotypes were grown at three locations over two consecutive crop years (2010 and 2011).

### 2.2. Locations

Three locations were selected to represent the wheat growing conditions of the Canadian Prairies. These were the Cereal Grain Research Centers of the Lethbridge (Alberta (AB)), Indian Head (Saskatchewan (SK)), and Portage la Prairie (Manitoba (MB)).

Environmental data conditions were obtained from Statistics Canada [16]. Location characteristics and soil composition data were obtained from Agriculture and Agri-Food Canada [17]. The mean temperature was lower in 2010 compared to 2011 across all locations, whereas total precipitation was higher in 2010 compared to 2011 across all locations.

### 2.3. Materials

Phenolic acids standards were purchased from Sigma-Aldrich (Milwaukee, Wisconsin, USA). All acids and organic solvents were obtained from Fisher Scientific (Whitby, Ontario, Canada). All chemicals used were of analytical grade.

### 2.4. Preparation of Free and Bound Phenolic Acid Extracts

The whole wheat samples were prepared as explained in a previous report [18]. Briefly, they were milled and passed through a 0.5 mm sieve screen using 14,000 rpm. The fine flour from each sample was stored at −20°C in the dark until further processing. Free and bound phenolic acid extractions were performed using liquid-liquid extraction and alkaline hydrolysis steps [19] as described before [18]. These extracts of free or bond phenolic acids were used in the cell culture experiments without further purification in order to keep sample degradation minimum and to mimic composition in the intestinal tract. It is expected that these extracts contain some nonpolar compounds other than the phenolic acids. It is expected that the amount of these compounds in the free phenolic extracts is low and would not interfere with the analysis of the glucose inhibition. However, it can be expected that hydrolysis products of a variety of cell wall polysaccharides in the fiber fraction may be present in the bound phenolic extracts and that these compounds could bind a fraction of the phenolics to render them inactive in the inhibition assays [18].

### 2.5. HPLC-PDA Analysis of Free and Bound Extracts

Phenolic acids of the free and bound fractions were identified by a reverse-phased performance liquid chromatography system (Waters 2695, Milford, MA, USA) equipped with a photodiode array detector (PDA) (Waters 996) and autosampler (717 plus, Waters, Milford, MA, USA) as described by Shamloo et al. [18].

### 2.6. Glucose Uptake Inhibition Activity Assays

#### 2.6.1. Inhibition of Glucose Uptake into Caco-2E Enterocyte Monolayers

The ability of wheat phenolic acid extracts to reduce cellular glucose uptake was investigated through the modified method described by Kwon et al. [10]. The model of confluent Caco-2 cell monolayers grown on 96-well plates was chosen because these previous studies indicated low bioavailability of dietary phenols; therefore, the main inhibitory action was expected to affect apical rather than basolateral transporters, which is captured by the chosen model.

To initiate the experiment, the confluent Caco-2 cell monolayers were rinsed 3 times with PBS and incubated in preincubation buffer (HEPES buffer with 5 mM glucose) for 30 minutes at 37°C. Transport experiments were initiated by replacing the buffer with 100 *µ*L HEPES buffer (pH 7.4 and glucose free), containing [^3^H] 2-deoxyglucose (5 mM in glucose-free HEPES buffer) and wheat extracts. The cells were incubated at room temperature for 15 minutes in the dark, and the transport experiment was stopped by adding 100 *µ*L of ice-cold preincubation buffer immediately after removal of transport buffer. Cells were washed with 100 *µ*L of preincubation buffer and then lysed with 60 *µ*L lysis buffer (20 mg SDS in 1 mL 0.2 M NaOH) and incubated at room temperature for 1 hour. 45 *µ*L aliquot of cell lysates was added to 5 ml scintillation cocktail, and the [^3^H] 2-deoxyglucose concentration was quantified by scintillation spectrometry. Protein content of the remaining cell lysates was determined using DC Protein Assay Kit (Bio-Rad, USA). The glucose uptake into early passages of Caco-2 cells (Caco-2E, passages 35–47) was expressed as counts per minute beta (cpma) per mg protein. Viability of cells and validity of the assay were demonstrated by the linearity of the uptake rates of glucose in the absence of wheat extracts.

### 2.7. Statistical Analysis

All cell culture data represent means of three independent experiments (three sets on different days), where three parallel transport experiments using three cell culture wells were performed. All data were analyzed using one-way analysis of variance (ANOVA) on a Minitab 14 Statistical Software (Minitab Inc., State college, PA, USA). Sample means were compared using Tukey HSD method, and significant differences were considered when *P* < 0.05. Correlations between wheat extract phenolic acid and flavonoid contents and inhibition capacity were done by Pearson's correlation test.

## 3. Results and Discussion

### 3.1. Genotype and Environmental Variation Influence on Phenolic Acids Levels

Phenolic acid (PA) contents in the free and bound fractions of the eight wheat varieties grown at different locations are listed in Tables 1 and 2, respectively. Free PA contents across all wheat genotypes grown during 2010 and 2011 ranged from 4.56 microgram per gram of dry matter (μg/g·dm) in Strongfield grown in Manitoba (MB) in 2010 to 40.98 *μ*g/g·dm in AC Barrie grown in Alberta (AB) in 2011. Contents of free PA were lower in 2010 than in 2011 for all genotypes except for AC Andrew (Table 1). Generally, wheat grown in AB in 2011 produced higher amounts of free PA; specifically, AC Barrie grown in AB contained the highest amount of free PA (40.98 *μ*g/g·dm) of all genotypes.

Bound PA levels of all genotypes grown during 2010 and 2011 ranged from 366.1 *μ*g/g dm (AC Navigator, MB, 2010) to 690.12 *μ*g/g dm (AC Crystal, AB, 2010). AC Crystal, AC Corrine, and AC Navigator (641.6 *μ*g/g·dm) grown in AB in 2010 and 2011 contained the highest amounts of bound PA, respectively.

Similar to previously reported data, the contribution of the genotype [20] on free phenolic acid levels in whole wheat is surpassed by the influence of environmental conditions[15, 21] reflected by the yearly variance in the presented data.

Bound phenolic acid levels were also more influenced by environment and less by genotype. However, bound PA in some genotypes (AC Andrew and Snowbird) showed lower sensitivity to environmental shifts being more stable in composition. This was in line with the results of Mpofu et al. [15]. The values reported for whole wheat bread correspond well with our data, where the highest level of total bound phenolic acids was 690 *μ*g/g.

### 3.2. Effect of Free and Bound Phenolic Acids on Glucose Uptake

**Figure 1:**
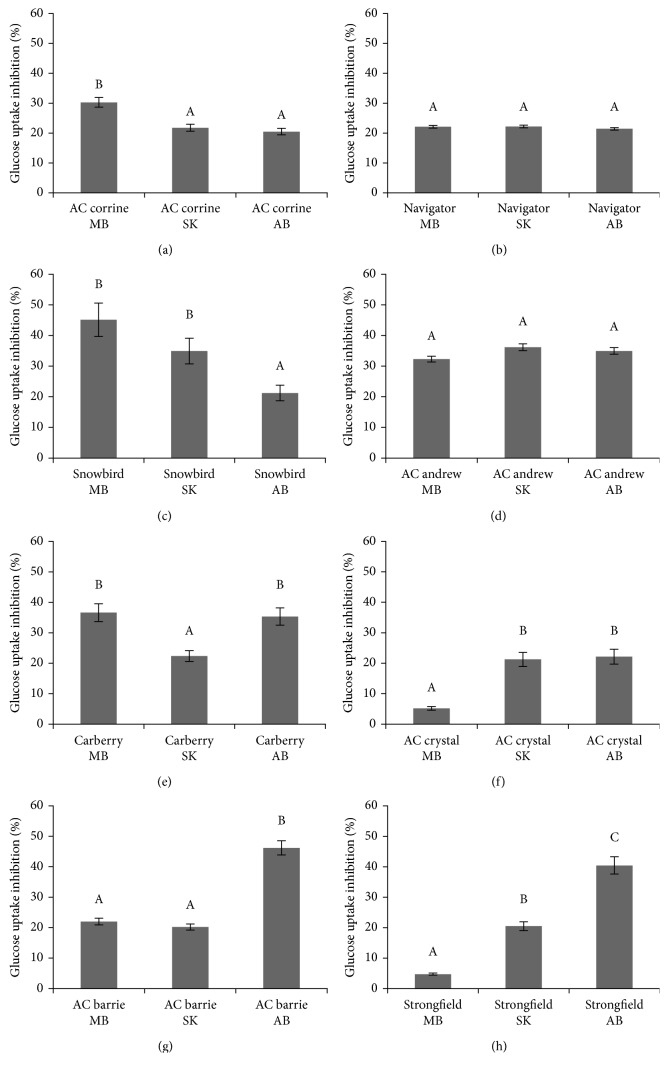
Relative inhibition of the uptake of [^3^H] 2-deoxyglucose into CaCo-2 monolayers caused by extracts of free phenolic acids obtained from eight wheat genotypes (a–h) grown in MB, SK, and AB over 2010 crop year. ^A,B,C^Different capital letter superscripts indicate significant differences (*P* < 0.05).

**Figure 2:**
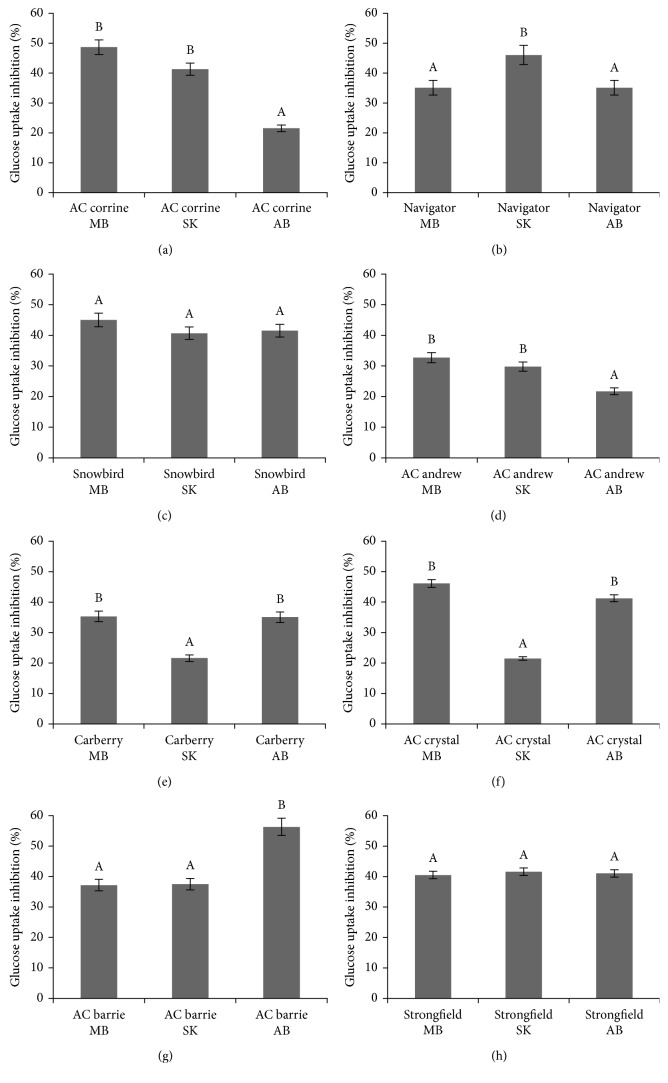
Relative inhibition of the uptake of [^3^H] 2-deoxyglucose into CaCo-2 monolayers caused by extracts of free phenolic acids obtained from eight wheat genotypes (a–h) grown in MB, SK, and AB over 2011 crop year. ^A,B,C^Different capital letter superscripts indicate significant differences (*P* < 0.05).

**Figure 3:**
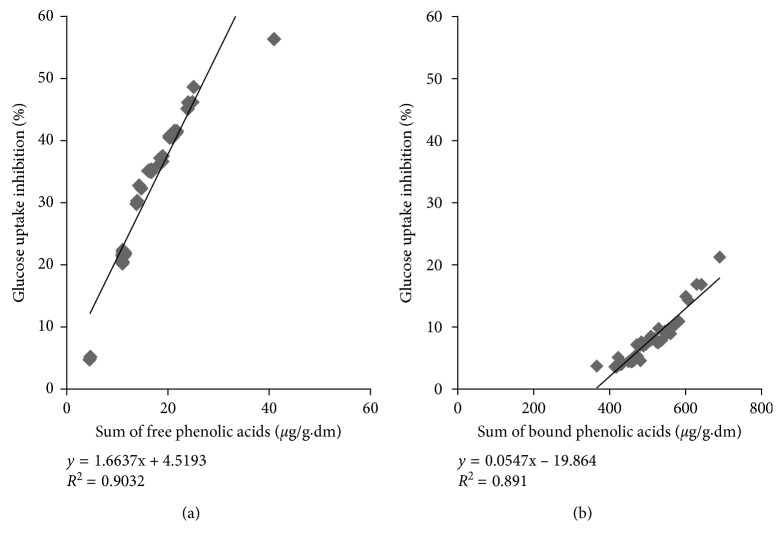
Correlation between the relative inhibition of glucose uptake into CaCo-2 monolayers and free phenolic acid (a) and bound phenolic acid (b) contents in extracts of eight wheat genotypes grown in MB, SK, and AB over 2011 crop year.

Free phenolic acid extracts of all whole wheat varieties grown at MB, SK, and AB over 2010 and 2011 inhibited glucose uptake in confluent Caco-2E monolayers as shown in Figures 1(a)–1(h) and Figures 2(a)–2(h), respectively. For the 2010 crop year, extracts from AC Barrie grown in AB showed the highest inhibition (46.18%) (Figure 1(g)), followed by Snowbird grown in MB (45.12%) (Figure 1(c)). For the 2011 crop year, again extracts from AC Barrie grown in AB showed the highest inhibitory potency (56.32%) (Figure 2(g)), followed by AC Corrine grown in MB (48.62%) (Figure 2(a)). The free phenolic acid content of all wheat genotypes positively correlated with the degree of inhibition of glucose uptake, as shown in Figure 3(a) (*R*^2^=0.903; *P* < 0.05).

**Figure 4:**
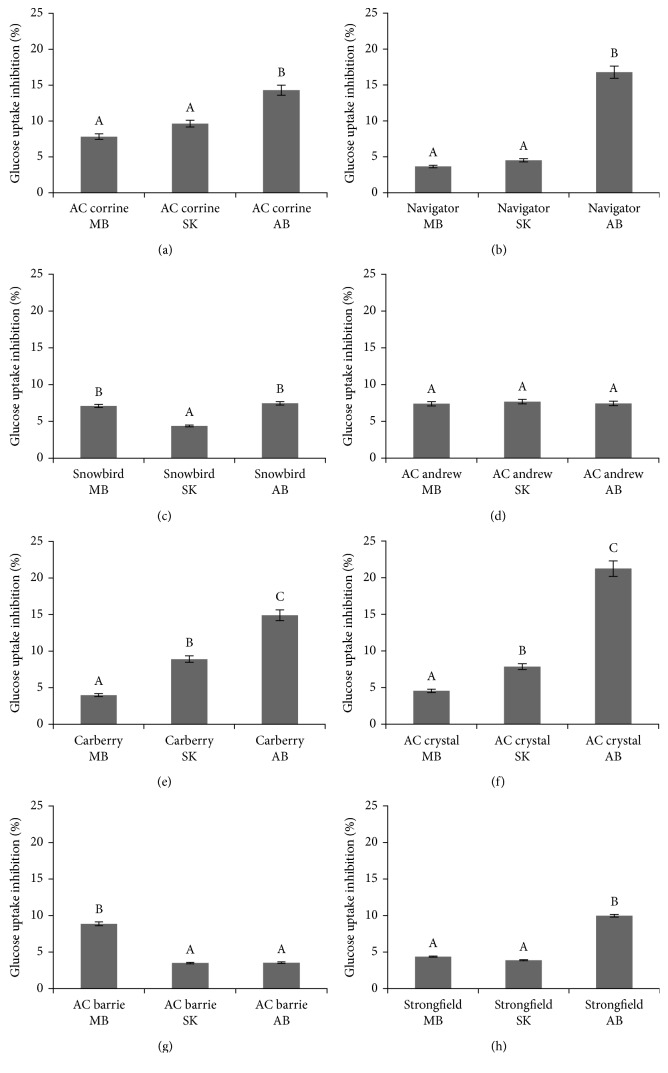
Relative inhibition of the uptake of [^3^H] 2-deoxyglucose into CaCo-2 monolayers caused by extracts of bond phenolic acids obtained from eight wheat genotypes (a–h) grown in MB, SK, and AB over 2010 crop year. ^A,B,C^Different capital letter superscripts indicate significant differences (*P* < 0.05).

**Figure 5:**
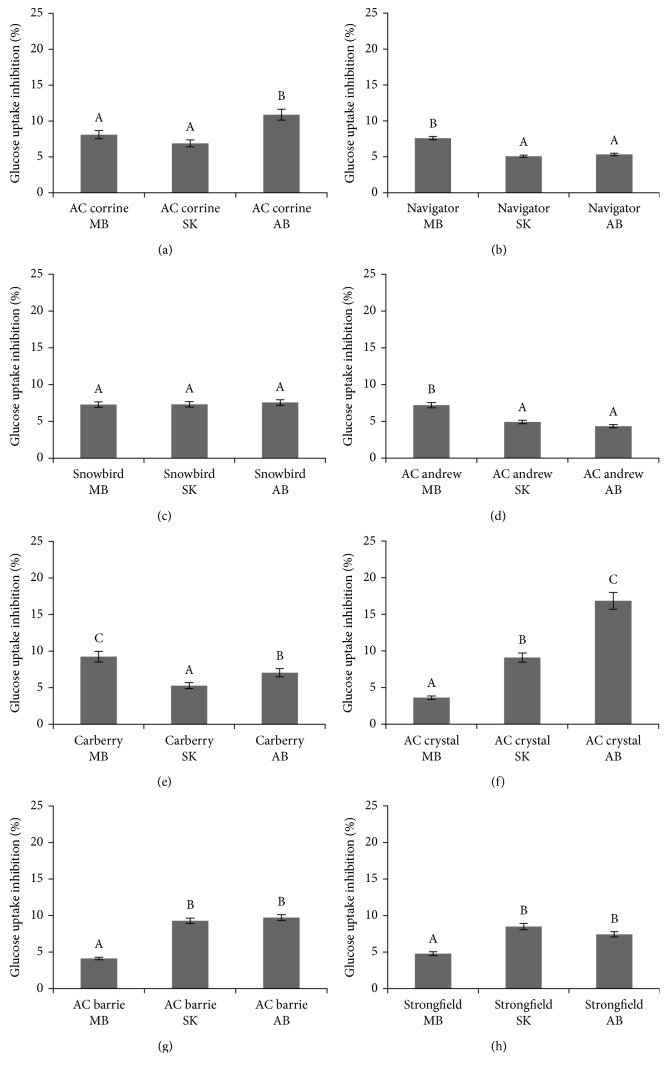
Relative inhibition of the uptake of [^3^H] 2-deoxyglucose into CaCo-2 monolayers caused by extracts of bond phenolic acids obtained from eight wheat genotypes (a–h) grown in MB, SK, and AB over 2011 crop year. ^A,B,C^Different capital letter superscripts indicate significant differences (*P* < 0.05).

Bound phenolic acid extracts of all wheat genotypes grown in MB, SK, and AB in 2010 and 2011 also inhibited the uptake of glucose in confluent Caco-2E monolayers, as shown in Figures 4(a)–4(h) and Figures 5(a)–5(h), respectively. The highest inhibitory potencies (21.22% and 16.78%) were observed for the extracts from AC Crystal and AC Navigator grown in AB in 2010 (Figures 4(f) and 4(b)), respectively. The degree of glucose uptake inhibitions positively correlated with the content of bound phenolic acids, as depicted in Figure 3(b) (*R*^2^=0.891; *P* < 0.05).

Extracts of free and bound phenolic acids inhibited glucose uptake in this Caco-2 model; however, the potency of the free phenolic acid extracts exceeded the potency of the extracts of bound phenolic acids. Free phenolic acids are fully bioaccessible, and some are bioavailable in the small intestine [22], while the bound phenolics are bound to the molecules of the fiber fraction and not fully bioaccessible. This might be reflected in our model, where we used extracts low in interfering fiber components in the free phenolic fraction where they exhibited their full potency. In contrast, the presence of hydrolyzed fiber constituents in the bound fraction could explain the reduced potency observed for these extracts. However, phenolic acids from both fractions can contribute to a blunting of cellular glucose uptake into small intestinal cells and therefore could contribute to the dampening of postprandial hyperglycemia.

The concentrations of total flavonoids in the glucose inhibition assays ranged between 0.5 and 2 *µ*g/ml for free phenolics and 35–50 *µ*g/ml for bound phenolics. Assuming an average molecular mass of 200 g/mol, the highest concentration of total free phenolic acids would have been 10 *µ*mol/l and from bound phenolic acid 250 *µ*mol/l. Assuming a stomach volume of one liter, the intake of 2 mg free phenolic acid or 35 mg of bound phenolic acids would achieve the concentrations used in our glucose inhibition assays. The most abundant compound in both free and bound phenolic acid extracts in wheat is ferulic acid, and it has been suggested that a daily dietary uptake of about 77 mg ferulic acid may suppress hyperglycemia [22]. Wheat bran is one of the main food sources of ferulic acid (5 mg/g) [23]. However, the daily intake of (free or bound) ferulic acid largely depends on the selection of cereal products. It had been estimated that whole wheat and refined wheat bread contain 330.1 *μ*g/g and 25.3 *μ*g/g of ferulic acid, respectively [21]. Considering the reported amounts of individual and whole phenolic acid in whole wheat, it is reasonable to assume that the human diet contains sufficient amounts to effectively inhibit glucose absorption. This might be reflected in the lower glycemic indices reported for whole wheat products compared to refined wheat products. As a consequence the consumption of whole wheat over refined products should be encouraged considering that the blood glucose blunt efficacy is largely lost through refinement.

## 4. Conclusions

The wheat genotype and growing conditions determine the content of free and bound phenolic acids. Levels of free and bound phenolic acids in extracts positively correlate with the inhibition of glucose uptake in a model of intestinal absorption.

## Figures and Tables

**Table 1:** Free phenolic acid contents (microgram per gram of dry matter) in the whole grain of 8 wheat varieties grown in three locations in 2010 and 2011 crop years.

Genotype	Year	Growing locations		
		MB	SK	AB
AC Corrine	2010	13.93 ± 0.98^A^	11.56 ± 1.27^A^	11.08 ± 1.09^A^
	2011	25.11 ± 2.92^B^	21.68 ± 2.65^B^	11.16 ± 1.82^A^
AC Navigator	2010	11.19 ± 1.07^A^	11.14 ± 1.67^A^	11.10 ± 2.93^A^
	2011	16.13 ± 1.81^B^	24.07 ± 2.94^B^	16.53 ± 2.87^B^
Snowbird	2010	23.91 ± 1.73^A^	16.79 ± 2.90^A^	11.17 ± 2.43^A^
	2011	23.98 ± 1.09^A^	20.28 ± 2.31^A^	21.79 ± 0.95^B^
AC Andrew	2010	14.75 ± 1.60^A^	18.34 ± 1.01^A^	16.62 ± 0.09^A^
	2011	14.34 ± 0.62^A^	13.81 ± 1.21^B^	11.24 ± 1.01^B^
Carberry	2010	18.93 ± 3.2^A^	11.10 ± 0.29^A^	16.31 ± 2.07^A^
	2011	16.72 ± 1.9^A^	11.15 ± 2.35^A^	16.55 ± 1.98^B^
AC Crystal	2010	4.68 ± 0.63^A^	11.14 ± 2.85^A^	11.49 ± 0.93^A^
	2011	24.03 ± 1.01^B^	11.42 ± 2.39^A^	21.43 ± 1.02^B^
AC Barrie	2010	11.12 ± 1.29^A^	11.06 ± 0.93^A^	24.83 ± 2.87^A^
	2011	18.51 ± 2.76^B^	18.92 ± 2.01^B^	40.98 ± 2.81^B^
Strongfield	2010	4.56 ± 2.9^A^	11.03 ± 1.76^A^	20.34 ± 2.09^A^
	2011	20.91 ± 1.87^B^	21.33 ± 0.98^B^	21.29 ± 2.76^A^

Values are given as mean ± SD from duplicate determinations.^A, B^Different superscript capital letters in the same column in the same dependent variable indicate significant difference (*P* < 0.05). MB = Manitoba; SK = Saskatchewan; AB = Alberta.

**Table 2:** Bound phenolic acid contents (microgram per gram of dry matter) in the whole grain of 8 wheat varieties grown in three locations in 2010 and 2011 crop years.

Genotype	Year	Growing locations		
		MB	SK	AB
AC Corrine	2010	537.41 ± 5.23^A^	555.51 ± 5.44^A^	607.17 ± 9.19^A^
	2011	519.54 ± 6.31^B^	489.23 ± 3.33^B^	582.34 ± 8.32^B^
AC Navigator	2010	366.11 ± 5.03^A^	463.47 ± 4.65^A^	641.6 ± 9.03^A^
	2011	522.78 ± 7.42^B^	422.61 ± 3.98^B^	467.28 ± 6.17^B^
Snowbird	2010	471.71 ± 6.54^A^	450.01 ± 5.87^A^	528.61 ± 5.54^A^
	2011	488.32 ± 7.30^A^	487.24 ± 2.63^A^	483.01 ± 4.59^B^
AC Andrew	2010	492.97 ± 5.20^A^	521.54 ± 4.93^A^	486.34 ± 8.98^A^
	2011	495.96 ± 4.82^A^	461.28 ± 5.75^B^	457.65 ± 6.46^B^
Carberry	2010	421.35 ± 7.17^A^	551.45 ± 5.43^A^	600.87 ± 7.34^A^
	2011	544.86 ± 8.43^B^	472.96 ± 4.54^B^	481.57 ± 8.22^B^
AC Crystal	2010	481.24 ± 9.32^A^	509.15 ± 6.38^A^	690.12 ± 7.56^A^
	2011	415.12 ± 6.82^B^	545.49 ± 10.43^B^	629.27 ± 6.41^B^
AC Barrie	2010	560.98 ± 8.38^A^	417.86 ± 7.77^A^	414.11 ± 5.89^A^
	2011	433.89 ± 5.16^B^	547.56 ± 6.09^B^	528.81 ± 7.94^B^
Strongfield	2010	459.11 ± 6.32^A^	430.32 ± 5.98^A^	566.96 ± 6.89^A^
	2011	458.68 ± 5.22^A^	509.01 ± 8.89^B^	525.81 ± 3.37^A^

Values are given as mean ± SD from duplicate determinations.^A, B^Different superscript capital letters in the same column in the same dependent variable indicate significant difference (*P* < 0.05). MB = Manitoba; SK = Saskatchewan; AB = Alberta.
